# Neutrophil extracellular traps in neonatal necrotizing enterocolitis: from developmental maturation to immunothrombotic injury

**DOI:** 10.3389/fimmu.2026.1972426

**Published:** 2026-10-20

**Authors:** Yang Chen, Mengru Zhu

**Affiliations:** Department of Neonatology, Children’s Medical Center, First Hospital of Jilin University, Changchun, China

**Keywords:** biomarkers, immunothrombosis, intestinal mucosal barrier, necrotizing enterocolitis, neutrophil extracellular traps, premature infants

## Abstract

Neonatal necrotizing enterocolitis (NEC) is best understood as a syndrome of intestinal necrosis arising in a developmentally susceptible host, with overlapping inflammatory, ischemic, infectious, cardiac-related, and transfusion-associated triggers. Neutrophil extracellular traps (NETs) are extracellular DNA networks decorated with histones, neutrophil elastase, myeloperoxidase, and other granule proteins. They may limit pathogen dissemination, yet persistent NET formation or impaired NET clearance can injure epithelium and microvascular endothelium and amplify immunothrombosis. This review focuses specifically on NETs in NEC. We first condense the general NEC background and then appraise human, animal, and mechanistic evidence with explicit attention to model system, intervention timing, NET detection method, infection burden, and NEC subtype. The neonatal NET-inhibitory factor (nNIF)/NET-maturation window is presented as a testable developmental hypothesis rather than an established quantitative rule. We synthesize biomarker and intervention literature and conclude that the translational question is not simply whether NETs are “good” or “bad”, but which infants, at which disease phase, defined by which combination of formation, clearance, platelet, endothelial, microbial, and imaging signals, may benefit from inhibition, clearance, or preservation of NET function.

## 1 Introduction

Necrotizing enterocolitis (NEC) occurs predominantly in premature and very-low-birth-weight infants and most often involves the terminal ileum and ascending colon, although severe disease may extend proximally or become pan-intestinal. Feeding intolerance, abdominal distension, gastric retention, bloody stools, ileus, and systemic instability are common, whereas pneumatosis intestinalis, portal venous gas, fixed loops, and free air are later or more specific imaging signs. Because early symptoms overlap with feeding intolerance, infectious enterocolitis, cow-milk protein-related enteropathy, and spontaneous intestinal perforation (SIP), the central clinical problem is not merely detecting inflammation, but identifying infants who will progress to irreversible necrosis (1–3).

The Bell staging system, first proposed to guide therapeutic decisions by clinical stage (7), remains useful for bedside communication, but it does not capture NEC heterogeneity. Contemporary clinical and translational literature increasingly distinguishes classical inflammatory NEC in very premature infants from ischemia-dominant disease, cardiac-related NEC associated with congenital heart disease and mesenteric venous congestion, transfusion-associated presentations, viral, or infection-triggered phenotypes, and localized perforation. Treating these entities as one homogeneous disease likely contributes to inconsistent replication of biomarker and intervention studies (2, 6, 8–10).

The clinical burden extends beyond acute survival. Surgical NEC and SIP are associated with growth failure, short bowel syndrome, intestinal failure-associated liver disease, recurrent infection, rehospitalization, and adverse neurodevelopmental outcomes. Recent core-outcome work therefore recommends endpoints that include death, surgical NEC, bowel resection, bacteremia, acute kidney injury, time to full enteral feeding, hospitalization, and later developmental follow-up rather than intestinal scores alone (11–16).

Recent advances include single-cell and spatial resolution of NEC tissue microenvironments (17, 18), evidence that epithelial injury is regulated by ferroptosis, autophagy-lysosomal dysfunction, and bile-acid/FXR pathways (19–24), and renewed attention to immune checkpoints such as macrophage α7nAChR/mTOR/NLRP3/interleukin (IL)-1β, triggering receptor expressed on myeloid cells 1 (TREM1), Th17/Treg balance, and disease tolerance (25–28). These findings do not replace the classical prematurity-feeding-microbiota-ischemia framework (29–31), but they refine the molecular nodes at which neutrophils may become decisive.

Neutrophil extracellular traps (NETs) sit at the intersection of these pathways. They are not simply a marker of neutrophil influx: their DNA scaffold concentrates proteases and cationic histones, while their surfaces support platelet adhesion, complement activation, and coagulation. The same structure can therefore be antimicrobial, thrombogenic, and cytotoxic depending on dose, location, duration, clearance, and host developmental stage. This review uses “NETs” for the extracellular structures and reserves “NET formation” or “neutrophil extracellular trap formation (NETosis)” for the process; “NET” is not used as a standalone noun.

Developmental maturation provides the context in which this review examines NETs. Neonatal neutrophils are not miniature adult neutrophils: term and preterm neonatal neutrophils generally show lower stimulus-induced NET formation than adult cells, with preterm cells lower than term cells under several stimulation conditions (52, 56–60). Umbilical cord blood contains neonatal NET-inhibitory factor (nNIF), a peptide derived from the carboxyl terminus of α1-antitrypsin, and placenta-derived HTRA1 can generate NET-inhibitory peptides by cleaving α1-antitrypsin (58, 61). In longitudinal observations of premature infants, stimulus-induced NET formation became detectable around postnatal day 3 and was relatively prominent between days 3 and 14, a maturational pattern that coincides with feeding advancement and microbial succession, the period in which NEC most often emerges (58, 61). The developmental regulation of NET formation, detailed in Section 3, is therefore not background material but the organizing premise of the NET-focused argument that follows.

## 2 NET biology, formation, clearance, and measurement: what can and cannot be inferred

### 2.1 Composition and functional logic

NETs were described as DNA-based extracellular traps that capture and kill microbes. Classical NETs contain decondensed chromatin, myeloperoxidase (MPO), neutrophil elastase (NE), cathepsin G, lactoferrin, defensins, and calprotectin. Their evolutionary logic is to immobilize pathogens and concentrate antimicrobial effectors, but the cost is exposure of host tissue to proteases, histones, and prothrombotic surfaces (32–36).

### 2.2 Pathways are stimulus- and context-dependent

Suicidal NET formation commonly involves NADPH oxidase-derived reactive oxygen species, NE and MPO translocation, chromatin decondensation, nuclear membrane rupture, and cell lysis. Peptidyl arginine deiminase 4 (PAD4)-mediated histone citrullination can promote decondensation, and citrullinated histone H3 (H3cit) is a useful but incomplete proxy for PAD4-dependent NET formation. However, PAD4-independent NET formation, vital NET release with preserved neutrophil migration, and mitochondrial DNA-containing traps have all been described. Consequently, H3cit positivity alone, MPO positivity alone, or extracellular DNA alone should not be equated with NETs (37–45).

### 2.3 Clearance is as important as formation

Deoxyribonuclease (DNase), macrophage phagocytosis, local proteolysis, complement regulators, and endothelial surfaces determine how long NETs persist. NETs, complement, and coagulation form a mutually reinforcing triangle: DNA scaffolds support platelet adhesion and thrombin generation, activated platelets and thrombin further activate neutrophils, and complement increases recruitment and permeability. In NEC, this triangle links mucosal infection, endothelial injury, microthrombosis, and systemic inflammation (23, 24, 46–49).

### 2.4 Measurement standards for NEC studies

Tissue studies should show extracellular DNA morphology with MPO or NE co-localization and, where possible, H3cit or other NET components, alongside spatial relation to neutrophil markers and injured epithelium/endothelium. Circulating cell-free DNA (cfDNA) is sensitive but nonspecific; nucleosomes indicate chromatin release; MPO-DNA and NE-DNA complexes are closer to NETs but can still reflect degranulation or non-NET DNA; H3cit is more specificbut may miss PAD4-independent NETs. Neonatal samples add constraints of low blood volume, hemolysis, timing relative to antibiotics and transfusion, and *in vitro* activation. Studies should report anticoagulant, processing time, freeze-thaw history, platform, gestational age, postnatal age, infection, antibiotics, feeding, transfusion, and timing of surgery (50–54).

For mechanistic interpretation, it is helpful to separate induction (microbial products, hypoxia, platelet signals), execution (ROS, NE/MPO, PAD4, calcium and cytoskeletal programs), and consequence (epithelial injury, endothelial activation/ferroptosis, platelet aggregation, coagulation, complement, and systemic spread). Interventions that block execution are not equivalent to interventions that clear already deposited NETs, and preventive blockade is not equivalent to treatment after injury is established (23, 37, 46, 55).

## 3 Developmental regulation of neonatal NETs formation and the proposed NEC time window

Neonatal neutrophils are not miniature adult neutrophils. Term and preterm neutrophils generally show lower NET formation after *in vitro* stimulation than adult neutrophils, with preterm cells lower than term cells in several stimulation conditions. This should not automatically be labeled a defect: limited NET formation during initial microbial colonization may reduce mucosal and pulmonary tissue injury while other antimicrobial functions mature (52, 56–60).

Yost and colleagues identified neonatal NET-inhibitory factor (nNIF) in umbilical cord blood, derived from the carboxyl terminus of α1-antitrypsin and reported that placenta-derived HTRA1 can cleave α1-antitrypsin to generate NET-inhibitory peptides. In longitudinal premature-infant observations, stimulus-induced NET formation became detectable around postnatal day 3, and was relatively prominent between days 3 and 14. These data are important but limited: they are largely *in vitro*/observational neonatal neutrophil studies, not a quantitative NEC-specific threshold, and they do not establish that nNIF decline causes NEC. We therefore advance the postnatal day 3–14 interval as a developmentally grounded heuristic, one that explains why NEC is uncommon at birth but clusters during feeding advancement and microbial succession, and that now requires prospective validation against clinical phenotypes (58, 61).

As a hypothesis rather than an established rule, the model predicts three phases: strong perinatal inhibition and low inducible NET formation; declining inhibition with rising intestinal antigenic and microbial stimulation; and, if homeostasis is restored, a regulated response. It becomes pathogenic only when superimposed on barrier injury, dysbiosis, hypoxia-ischemia, platelet activation, or impaired clearance (**Figure 1**). Infection models show that nNIF, PAD4 inhibition, or DNase can improve survival in peritonitis and polymicrobial sepsis, but these are not NEC models and cannot define the therapeutic window in intestinal necrosis (58, 61–64).

**Figure 1:**
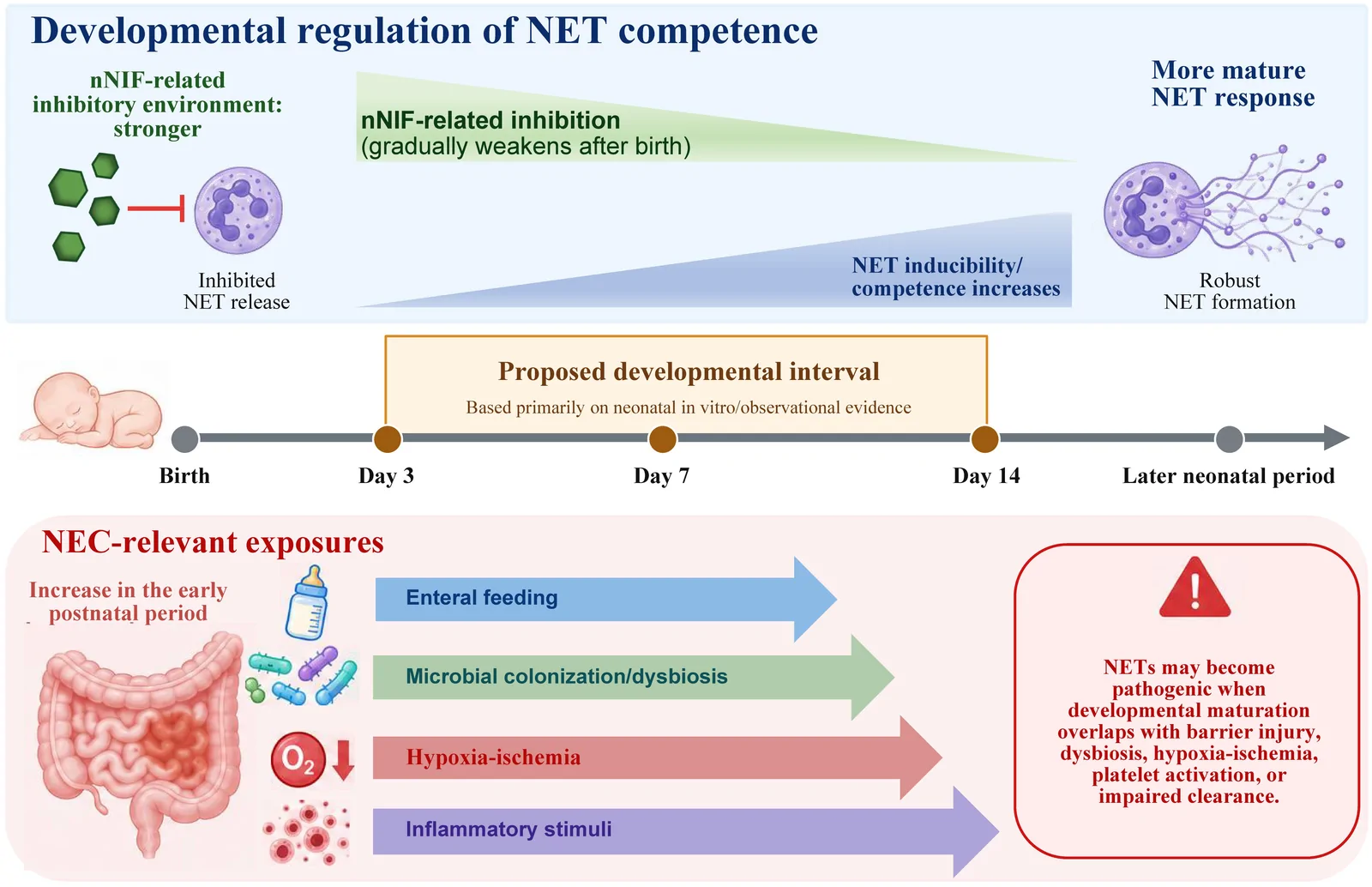
Conceptual model of developmental NET regulation and NEC-relevant exposures. After birth, nNIF-related inhibition gradually weakens as neutrophil NET competence matures, while enteral feeding, microbial colonization, hypoxia-ischemia, and inflammatory stimuli increase. The proposed postnatal day 3–14 interval is based primarily on limited neonatal *in vitro* and observational evidence and should be regarded as a hypothesis-guided developmental window rather than a validated quantitative threshold for NEC susceptibility.

Gestational age, postnatal age, birth weight, delivery mode, chorioamnionitis, perinatal infection, antibiotics, nutrition, transfusion, and platelet-neutrophil interactions should be treated as covariates rather than background noise. In particular, grouping by gestational age without correcting for postnatal age can mistake developmental maturation for disease biology (52, 60, 65, 66).

## 4 Evidence that NETs participate in NEC: convergent, conflicting, and subtype-dependent

### 4.1 Human tissues, blood, and fecal evidence

NEC surgical tissues show neutrophil infiltration with extracellular DNA, MPO/NE, and H3cit co-localization in injured mucosa. Fecal calprotectin is elevated around clinical onset, and has been localized to NET-like structures, suggesting that fecal calprotectin reflects both neutrophil influx and NET release. Human and rodent studies also report elevated serum cfDNA, nucleosomes, MPO-DNA/NE-DNA complexes, or H3cit in NEC, with correlations to severity in some cohorts. These data establish association, and plausibility, but surgical specimens usually represent late disease and cannot prove temporality; circulating markers remain vulnerable to hemolysis, necrosis-associated DNA release, degranulation, and non-NET sources (50, 51, 53, 54).

### 4.2 Animal intervention evidence is directionally inconsistent, and that is informative

In lipopolysaccharide (LPS)/formula/hypoxia mouse NEC models, PAD inhibition reduced mortality and tissue injury, while systemic DNase1 degraded extracellular DNA and reduced inflammation, oxidative stress, epithelial injury, and mortality (54, 67). By contrast, in a dithizone/Klebsiella model with prominent bacterial translocation, NET inhibition increased bacteremia, systemic inflammation, organ injury, and mortality; and in Citrobacter rodentium infection, PAD4-dependent NETs were required for mucosal clearance. These results are not mutually exclusive: they map onto different infection burdens, barrier states, and timing of intervention (68, 69).

### 4.3 Recent mechanistic studies place NETs within immunothrombosis

Recent work has moved the field from NETs are present to which cellular circuits do NETs execute? Cluster of differentiation 177 (CD177)+ neutrophil-platelet aggregates contribute to thromboinflammation via NETs in NEC; platelet-derived exosomes induce NET formation through a pathway attenuated by gasdermin D (GSDMD) inhibition; inositol hexakisphosphate kinase 1 (IP6K1)-dependent platelet polyphosphates regulate neutrophil-platelet aggregates; and IL-40 deficiency inhibits NETosis and ameliorates experimental injury. Together with endothelial ferroptosis and chromatin-alarmin studies, these findings position NETs as an effector module linking neutrophils, platelets, endothelium, complement, and coagulation rather than as an isolated biomarker (23–27, 70, 71). **Table 1** summarizes the model systems, NET interventions, detection methods, intervention timing, main outcomes, and principal limitations of the representative human and animal studies discussed in Sections 4.1 to 4.3.

**Table 1:** Critical comparison of representative human and animal evidence linking NETs to NEC.

Study/model	Stimulus/context	NET intervention	Timing	NET readout	Main outcome	Interpretation and limitation
Vincent/Klinke mouse NEC; Sci Rep 2018 (54)	LPS + formula + hypoxia	PAD inhibition	Preventive/therapeutic in model protocol	Tissue H3cit/MPO; serum cfDNA/nucleosomes	Reduced NEC manifestation (4/10 vs 5/7 with severe NEC, p=0.003) and day-10 mortality (p=0.0129; day-6 survival 7/14 NEC vs 10/14 with PAD inhibition); serum cfDNA correlated with NEC score (r=0.53, p=0.001) and Chiu score (r=0.56, p<0.001). Total 76 mice; human NEC tissue n=9	Supports pathogenic NETs in inflammatory-ischemic model; clinical samples mostly late; cfDNA nonspecific
Klinke/Vincent DNase mouse NEC; J Surg Res 2019 (67)	LPS + formula + hypoxia	Systemic DNase1	After injury induction	Extracellular DNA degradation; cfDNA/H3cit/NE/MPO/TLR4	N=44 neonatal mice; DNase1 i.p. from day 5 for 72 h reduced NEC rates (44% vs 86%, P = 0.029) and improved survival (65% vs 35%, P = 0.01), with lower cfDNA, Chiu score, MDA, GPx, NE, MPO, H3cit, and TLR4	Targets deposited DNA, not formation; does not remove histones/proteases; timing critical
Chaaban dithizone/Klebsiella NEC-like model; J Cell Mol Med 2021 (68)	Chemical injury + live Gram-negative bacteria	NET inhibition	Around bacterial challenge	NET-associated signals	Cl-amidine (40 mg/kg, pan-PAD inhibitor) given 30 min before and 3 h after Klebsiella gavage nearly doubled mortality (94.7% vs 47.6%, p<0.0001) and increased bacteremia at 1 h (p=0.0074); intestinal injury scores did not differ between NEC and NEC+Cl-amidine groups, supporting a defect in bacterial clearance rather than direct tissue protection	Shows protective NETs when translocation risk is high; not equivalent to classical hypoxia/LPS NEC
Saha Citrobacter rodentium; Mucosal Immunol 2019 (69)	Attaching/effacing infection	PAD4 deficiency or DNase	During infection	PAD4-dependent NETs	Pad4^−/−^ adult mice (8 weeks; histology n=6–8): CR colonization peaked at days 11–14 (vs clearance by day 14 in WT), with aggravated colonic hyperplasia, goblet cell depletion, and crypt apoptosis; DNase I (n=6) and Cl-amidine recapitulated the phenotype. No mortality endpoint; adult, non-NEC model	Mucosal defense context differs from NEC necrosis; supports organ- and phase-specific effects
CD177^+^ neutrophil-platelet aggregates; Nat Commun 2026 (25)	Human tissue/model mechanistic	Not a treatment trial	Observational/mechanistic	CD177^+^ aggregates, NETs, thromboinflammation	Neonatal mice: histology/flow n=12 per group, survival n=24 per group; CD177^−/−^ or myeloid-specific CD177^KO^ reduced intestinal NPAs and NETs and improved survival; DNase I or PAD4i (n=12/group) reduced histopathology scores; prophylactic LMWH disrupted NPAs, lowered NET burden, and improved survival. Human NEC: CD177^+^ NPA levels correlated with severity and thrombotic biomarkers (n=14, Spearman)	Strong mechanistic clue; requires prospective stratification and causal timing
Platelet exosome/GSDMD; Cell Commun Signal 2026 (26)	Platelet-derived exosomes	GSDMD inhibition	Mechanistic blockade	NET formation induced by exosomes	5-day-old C57BL/6 pups (formula gavage + enteral LPS 5 mg/kg + hypoxia/cold stress); disulfiram reduced platelet caspase-1 activation, NET levels, MPO/ROS, IL-6 and IL-1β, ameliorated villus necrosis, and significantly improved survival; GSDMD^−/−^ pups phenocopied. Mechanism: mitochondrial ROS and ox-mtDNA link platelet pyroptosis to NET formation	Identifies upstream node; clinical safety in neonates unknown
IP6K1-polyphosphate axis; Inflamm Res 2026 (70)	Platelet short-chain polyphosphates/NPA	IP6K1 inhibition	Experimental NEC	Neutrophil-platelet aggregates	Human NEC: elevated circulating NPAs and anticardiolipin IgG, reduced platelet counts with pyroptosis as dominant platelet death mode. Neonatal mouse NEC: Ip6k1^−/−^ and TNP (IP6K1 inhibitor) attenuated NPA formation, neutrophil infiltration, and NET release, improving survival and reducing severity scores; exogenous polyP rescued NPA formation; P-selectin neutralization reduced both NPAs and NETs	Supports selective immunothrombosis targeting; not direct NET quantification of all pathways
IL-40 deficiency; Cell Commun Signal 2026 (71)	Experimental NEC	IL-40 deficiency	Genetic/developmental	NETosis	Human NEC (n=8; surgical controls n=5): plasma IL-40 correlated with SOFA score (r=0.9259, P<0.0001) and MPO-DNA with SOFA (r=0.678, P = 0.0007). IL-40^−/−^ neonatal mice: higher survival, lower histopathology scores, reduced dsDNA, MPO-DNA, and H3Cit, and lower bacterial burden and plasma LPS	Cytokine-NET link; causality and reversibility need confirmation

TLR4, Toll-like receptor 4.

### 4.4 A causal-inference and reproducibility framework

Evidence should be graded by whether NET signals precede necrosis, track with subsequent severity, localize to injured epithelium/endothelium/platelet microthrombi, and are altered by interventions that change outcomes in more than one model. At present, human studies satisfy association and localization; animal studies satisfy several intervention criteria; and recent multi-omics/mechanistic studies strengthen biological plausibility. The weakest links are standardization, subtype stratification, prospective early sampling, and neonatal safety (18, 50, 51, 62, 72–77).

Reproducibility requires reporting of animal age and sex, feeding composition, hypoxia intensity, LPS dose, bacterial load, antibiotic exposure, intervention timing, endpoints, NET quantification, platelet/endothelial/complement/coagulation measures, bacterial translocation, and systemic organ outcomes. A survival benefit without bacterial-load data cannot distinguish infection control from tissue protection; a tissue-staining study without functional outcomes cannot establish clinical relevance (74, 75).

### 4.5 NEC subtype heterogeneity should be built into the design

NET biology is unlikely to be identical across NEC phenotypes. In classical inflammatory NEC, dysbiosis, TLR4 signaling, and neutrophil recruitment may dominate, making histone/protease injury and immunothrombosis more plausible. In ischemia-dominant or cardiac-related NEC, endothelial activation, venous congestion, and platelet-neutrophil aggregates may be upstream, with NETs acting as amplification rather than initiation. In transfusion-associated or viral-triggered presentations, immune complex-like signals, complement, and disease tolerance may modify NET formation. SIP and focal perforation should be analyzed separately because localized perforation with mild systemic inflammation may not share the same NET biology as segmental necrosis (2, 4–6, 9, 10, 16, 25, 26, 70, 78, 79).

**Table 2** should be read as a set of testable priorities rather than established conclusions. The intervention implications column in particular reflects biological reasoning from the upstream drivers and NET position columns, not clinical evidence, and no NET-directed therapy has been tested in any subtype-stratified human study. The purpose of the table is to make the stratification logic explicit so that future studies can confirm or refute each cell.

**Table 2:** Proposed NET-biology framework across NEC subtypes.

Subtype	Dominant upstream drivers	Likely NET position	Priority markers	Intervention implication
Classical inflammatory NEC (very preterm)	Dysbiosis, TLR4 signaling, feeding stress	Execution module of histone/protease injury and immunothrombosis	Tissue H3cit/MPO/NE co-localization; MPO-DNA, H3cit, fecal calprotectin	PAD4/DNase window plausible after bacterial control
Ischemia-dominant/cardiac-related NEC	Mesenteric hypoperfusion, venous congestion, platelet activation	Amplification of endothelial injury and microthrombosis rather than initiation	Platelet count/trajectory, CD177^+^ aggregates, mesenteric Doppler flow	Prefer platelet/endothelial nodes (IP6K1, TREM1) over global NET blockade
Transfusion-associated/viral-triggered	Immune-complex-like signals, complement, altered disease tolerance	Modulated by complement and tolerance state; contribution least established	Complement fragments, extracellular cold-inducible RNA-binding protein, NET panel	Exploratory only; no NET-directed inference yet
SIP/focal perforation	Localized wall stress, often minimal systemic inflammation	Probably distinct from segmental necrosis; do not pool with NEC	Perforation site histology, low systemic NET panel	Analyze separately in all NET studies

A practical stratification set includes: etiologic subtype; Bell stage and imaging/ultrasound blood flow; gestational and postnatal age; feeding and human-milk exposure; antibiotic and transfusion history; documented infection; congenital heart disease and mesenteric perfusion; platelet trajectory; and a NET panel combining tissue co-localization when tissue is available with cfDNA/nucleosomes, MPO-DNA/NE-DNA, H3cit, and fecal calprotectin. Randomized or interventional studies should not enroll by Bell stage alone; they should enrich for a prespecified NET-high-burden phenotype and include bacteremia and organ injury as safety endpoints (4, 5, 15, 51, 53, 54, 78).

## 5 The double-edged effect of NETs in NEC: a context-dependent model

**Figure 2** depicts the context-dependent model of NET action in NEC, in which the net effect of NETs shifts with infection burden, disease stage, developmental age, and clearance capacity.

**Figure 2:**
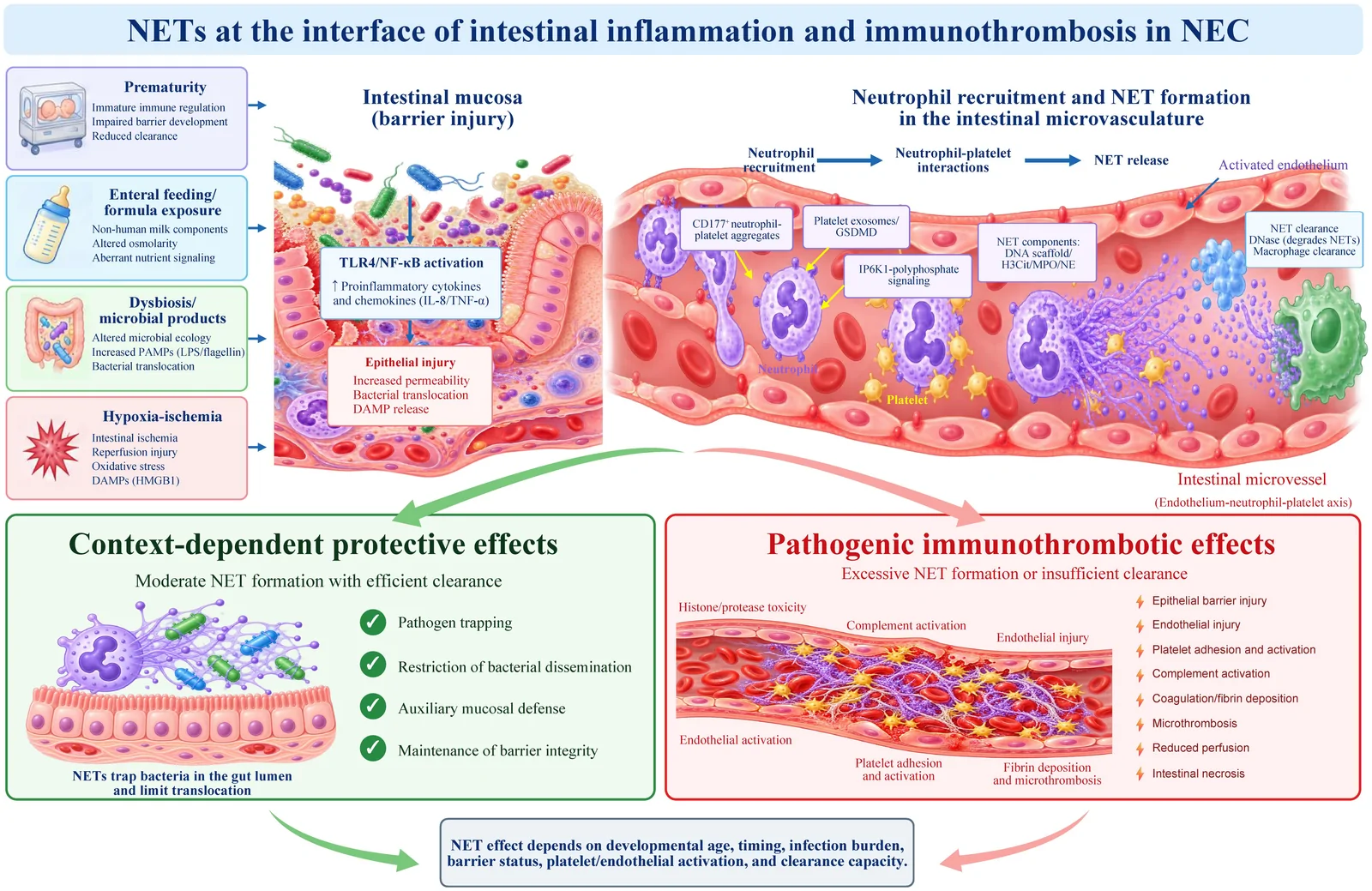
NETs in NEC: a context dependent model. Prematurity, feeding, dysbiosis, and hypoxia ischemia promote barrier injury, neutrophil recruitment, and NET formation. CD177+ aggregates, platelet exosome GSDMD signaling, and IP6K1 polyphosphates amplify thromboinflammation. Moderate NETs with effective clearance limit bacterial spread, whereas excessive NETs injure epithelium and endothelium and promote microthrombosis. The net effect depends on infection burden, disease stage, developmental age, and clearance capacity.

Protective effects are most plausible early, when bacterial load is high and epithelial repair is incomplete. DNA webs and concentrated MPO, NE, lactoferrin, and calprotectin can limit microbial spread, while controlled local fibrin and platelet responses may wall off infection. The same NETs become pro-damage when histones increase membrane permeability, NE/MPO degrade junctional and matrix proteins, extracellular DNA acts as a damage-associated molecular pattern, and DNA scaffolds accelerate platelet adhesion and thrombin generation (23, 24, 34–36, 46, 47, 55). **Table 3** translates this context-dependent biology into clinical implications and monitoring priorities across four characteristic scenarios.

**Table 3:** Context-dependent interpretation of NET protective versus injurious effects.

Context	Likely dominant NET biology	Potential clinical implication	Key monitoring signals
High bacterial load/translocation risk	NET capture and local antimicrobial concentration; immunothrombotic sealing	Avoid indiscriminate formation blockade; prioritize infection source control and antibiotics	Cultures, lactate, platelet count, perfusion, ultrasound blood flow, NET panel trend
Barrier injured; infection controlled	Persistent histone/protease injury; delayed clearance	Therapeutic window for DNase or short-course selective NET inhibition may exist	Falling bacterial burden but rising cfDNA/H3cit/MPO-DNA, ongoing platelet consumption
Ischemia/cardiac-related phenotype	Endothelial activation, platelet-neutrophil aggregates, microthrombosis	Evaluate perfusion and immunothrombosis nodes; avoid equating with classical inflammatory NEC	Echocardiography/perfusion status, mesenteric Doppler, CD177/platelet aggregates where available
Late/postoperative recovery	Necrosis-derived DNA, ongoing DAMP release, repair-versus-inflammation balance	Clearance and barrier repair may matter more than blocking formation	Enteral tolerance, inflammatory trajectory, renal/pulmonary injury, neurodevelopmental follow-up

Bidirectional microbiota-NET interactions should also be recognized. Microbial products can induce NETs; NETs can kill or reshape bacteria; and tolerant organisms may exploit DNA webs. Adult inflammatory bowel disease and ischemia-reperfusion studies are useful mechanistically but should not be directly mapped onto NEC because neonatal barrier development, colonization dynamics, platelet reserves, and nNIF regulation are distinct (69, 80–85).

## 6 Biomarkers: dynamic, multimodal, age-corrected, and subtype-aware

No single NET marker is sufficient for NEC diagnosis. The most defensible strategy is a longitudinal panel embedded in clinical care: feeding events, and gastric residuals; abdominal examination and circumference; temperature and circulatory support; platelets, C-reactive protein, lactate, renal function; imaging including ultrasound assessment of wall thickness, perfusion, portal venous gas, and fluid; and NET-related markers such as cfDNA, nucleosomes, MPO-DNA/NE-DNA, H3cit, and fecal calprotectin. Rising NET markers together with falling platelets, increasing lactate, and reduced intestinal wall blood flow are more informative than any isolated value (4, 5, 51, 53, 54, 76, 78). **Table 4** summarizes specimen type, potential indication, strengths, and main limitations of each NET-related marker and auxiliary assessment technology.

**Table 4:** NEC-relevant NET biomarkers and auxiliary assessment technologies.

Marker/technology	Sample	What it may indicate	Strengths	Main limitations
cfDNA/nucleosomes	Serum/plasma	Cell death, chromatin release, possible NETs	Easy, dynamic	Nonspecific; hemolysis, necrosis, apoptosis, platelets
H3cit	Blood/tissue	PAD4-dependent histone citrullination	Mechanistically specific for one NET pathway	Misses PAD4-independent NETs; timing sensitive
MPO-DNA/NE-DNA complexes	Blood	Granule proteins complexed with extracellular DNA	Closer to NETs than cfDNA	Degranulation plus non-NET DNA; assay/platform variability
Tissue DNA-MPO/NE-H3cit co-localization	Surgical/biopsy tissue	Local NET structures and spatial relationship to injury	Strong spatial evidence	Usually late disease; sampling and interpretation bias
Fecal calprotectin	Stool	Intestinal neutrophil influx; partially NET-derived	Noninvasive, repeatable	Affected by gestational age, feeding, non-specific enterocolitis
Ultrasound/POCUS and emerging optical/AI tools	Bedside imaging	Wall thickness, blood flow, portal venous gas, fluid; tissue oxygenation	Repeatable, can precede radiographic necrosis	Operator/device dependence; requires validation and NET correlation
Multi-omics + machine learning	Blood/stool/imaging/clinical	Microbial, metabolic, immune, and tissue-injury trajectories	May reveal pre-symptomatic risk	Small cohorts, variable definitions, external validation limited

A neonatal sepsis study caution is directly relevant: cfDNA may add some discrimination early, whereas MPO-DNA, NE-DNA, and H3cit did not consistently outperform conventional inflammatory markers and may reflect developmental degranulation rather than NETs. This argues against direct transplantation of adult NET panels and for reference ranges by gestational/postnatal age, standardized handling, and repeated sampling (51, 52, 60).

Metabolomic and microRNA signals, including bile-acid imbalance, short-chain fatty acid and tryptophan metabolites, and volatile organic compounds (77, 86, 87), together with transcriptomic and epigenetic regulators of epithelial death (56, 57, 70, 88), may improve risk prediction when combined with NET measures. Future models should predefine variables, correct for antibiotics and feeding, report calibration and decision curves, and undergo external validation in distinct gestational-age and center populations (18, 78, 85, 89, 90).

## 7 Intervention strategies: preserve defense, limit amplification, enhance clearance

NET-targeted strategies must remain subordinate to proven prevention and treatment: human milk when available, cautious feeding advancement, stewardship of antibiotics, infection control, individualized probiotic decisions in extremely premature infants, timely surgical consultation, and standardized perioperative care. Trials such as STAT for enterostomy versus primary anastomosis illustrate why procedure and outcome standardization matter for interpretation (91–94).

PAD4 inhibitors block histone citrullination and NET formation and have shown benefit in inflammatory-ischemic NEC models. Their risk is greatest when infection is not controlled or when PAD4-independent host defense is needed. Selectivity, pharmacokinetics, hematopoietic effects, and long-term developmental safety in neonates remain unresolved (41, 42, 54, 95). DNase1 targets already deposited extracellular DNA and may preserve early neutrophil defense while reducing persistent scaffold-mediated injury, but it does not neutralize histones or proteases and may clear mitochondrial or pathogen DNA with unpredictable effects (46, 55, 67). nNIF and related peptides are attractive because they mimic an endogenous neonatal regulatory mechanism, but NEC-specific efficacy, bacterial translocation risk, gestational-age window, and dosing remain preclinical questions (58, 61, 63, 64).

Upstream and parallel nodes may be safer than global NET suppression: GSDMD inhibition to reduce platelet-exosome-induced NET formation, IP6K1 inhibition to limit platelet polyphosphate-driven neutrophil-platelet aggregates, TREM1 modulation, anti-extracellular cold-inducible RNA-binding protein strategies, endothelial ferroptosis mitigation, and barrier repair with human-milk components or defined metabolites. These approaches should be tested as mechanisms within the same subtype- and timing-stratified framework rather than as interchangeable “anti-NET” drugs (21, 26, 27, 70, 96–106). **Table 5** summarizes the action node, evidence status, potential advantages, and key translational questions of each strategy.

**Table 5:** Translational prospects and boundaries of NETs- directed or NET-adjacent strategies.

Strategy	Action node	Evidence status	Potential advantage	Key translational question
PAD4 inhibition	Histone citrullination/NET formation	Benefit in LPS-hypoxia NEC models; harm possible in high-translocation models	Direct blockade of a defined execution step	When is infection controlled enough to inhibit formation without losing mucosal defense?
DNase/extracellular DNA clearance	Deposited DNA scaffold	Improved outcomes in a mouse NEC model	Preserves formation while limiting persistence	Can DNA clearance be timed after bacteremia risk falls and before irreversible necrosis?
nNIF-like peptides	Terminal NET formation steps	Peritonitis/sepsis models; NEC-specific data lacking	Physiologically aligned with neonatal endogenous regulation	Does supplementation increase translocation in real NEC subtypes?
Platelet/GSDMD/IP6K1/TREM1 nodes	Immunothrombosis and inflammatory amplification	Emerging NEC mechanism studies	More selective than global neutrophil suppression	Which node is causal in which subtype and phase?
Microecological/barrier repair	Upstream triggers: dysbiosis, metabolites, epithelial injury	Human milk components, defined metabolites, fecal filtrate/virome studies	Prevention-aligned and potentially safer	Are NET burden and epithelial repair measured as intermediate outcomes?

Negative results are informative. The observation that NET inhibition worsens bacteremia and mortality in high-translocation models should set safety boundaries for clinical trials: bacteremia, meningitis, fungal infection, wound healing, thrombosis/bleeding, liver and kidney injury, and neurodevelopment must be monitored. Preventive blockade in infants without a NET-high phenotype is biologically implausible and ethically difficult; late clearance without infection control may also fail. A realistic pathway is observational threshold definition, then timing experiments in at least two complementary models, then pharmacokinetic/safety work, and only then small stratified efficacy trials (15, 107–110). Taken together, these findings support a staged translational framework in which NET formation, persistence and clearance, and downstream immunothrombotic injury are assessed using complementary biomarkers and linked to mechanism-specific intervention strategies (**Figure 3**).

**Figure 3:**
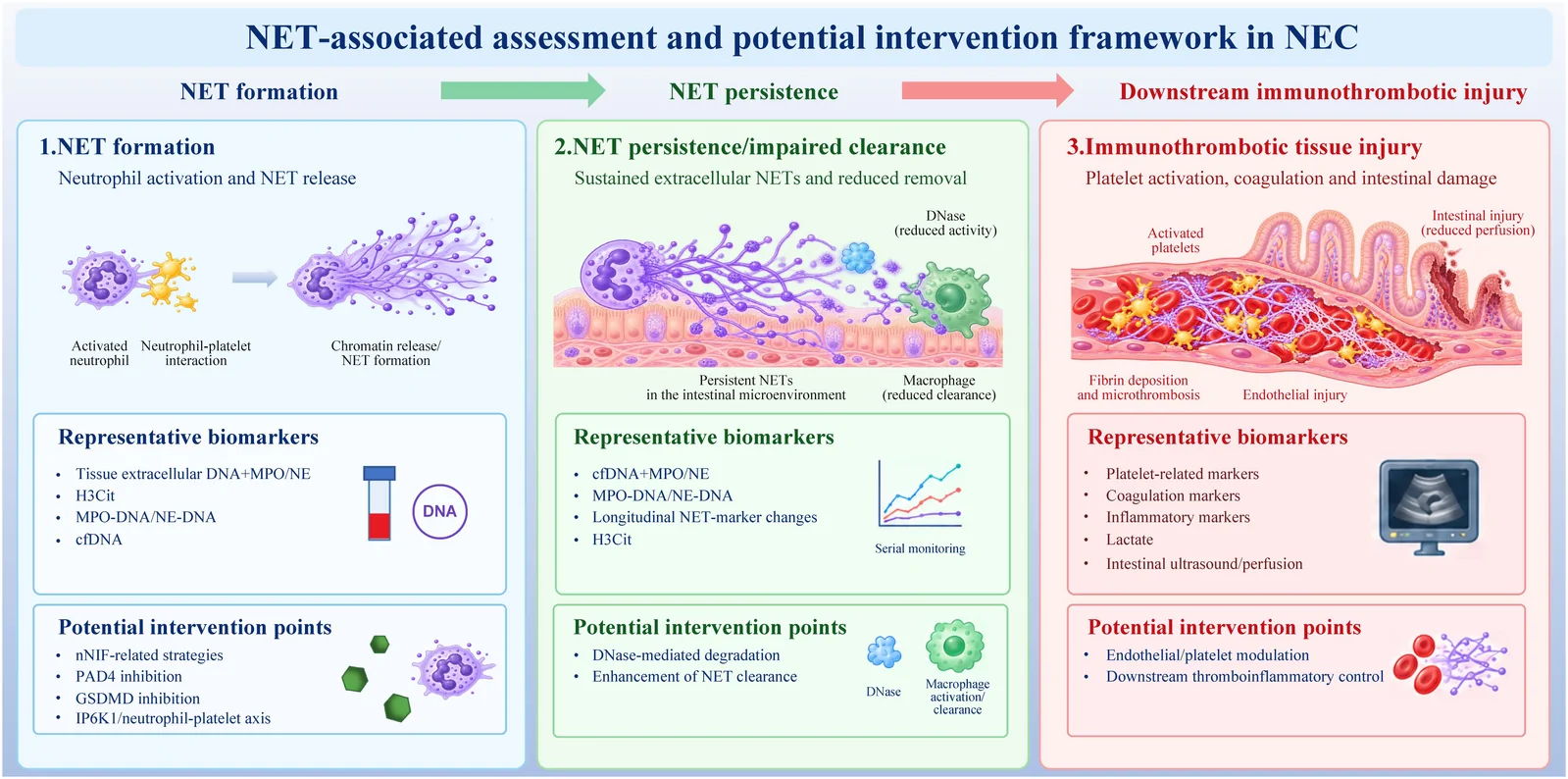
NET-associated assessment and potential intervention framework in NEC. NET-associated processes in NEC are conceptually divided into three stages: NET formation, NET persistence or impaired clearance, and downstream immunothrombotic injury. Representative markers include tissue extracellular DNA with MPO/NE colocalization, H3cit, MPO–DNA/NE–DNA complexes, cfDNA, platelet- and coagulation-related markers, inflammatory markers, and intestinal perfusion assessment. Potential intervention points include nNIF-related strategies, PAD4 or GSDMD inhibition, modulation of the IP6K1/neutrophil–platelet axis, DNase-mediated degradation, enhancement of macrophage-dependent clearance, and downstream endothelial or platelet modulation.

## 8 Future directions and critical gaps

Priority gaps are now narrower than a general call for more NET studies. First, detection must be standardized and age-corrected, with simultaneous assessment of formation and clearance. Second, NEC subtypes and SIP must be separated at enrollment and analysis. Third, prospective cohorts should test whether NET trajectories precede imaging-defined necrosis and predict surgical NEC, death, acute kidney injury, bacteremia, and neurodevelopmental outcome, and should specifically test the hypothesized postnatal day 3 to 14 window as a stratification variable rather than an established quantitative threshold. Fourth, animal and organoid work should define the sequence from platelet/endothelial activation to NET formation to epithelial necrosis and should compare block-formation versus clear-deposited-DNA strategies after bacterial control. Finally, neonatal drug development must include infection challenge, growth, immune maturation, coagulation, and developmental safety endpoints. Detailed protocols belong in grant applications; reviews should now emphasize these falsifiable boundaries (18, 50, 51, 62, 72–75, 107, 108).

## 9 Conclusion

NETs are biologically plausible and experimentally supported participants in NEC, but they are neither uniformly protective nor uniformly pathogenic. The field’s translational task is to identify measurable high-NET-burden phenotypes within defined NEC subtypes, determine when formation has shifted from defense to injury, and test interventions that inhibit formation, clear deposited NETs, or interrupt platelet-endothelial amplification only after infection control is secure. As summarized in **Table 2** and the subtype framework in Section 4.5, the balance between NET-mediated defense and NET-driven injury shifts with etiologic subtype, infection burden, disease phase, and clearance capacity; stratification along these axes, rather than unselected suppression, offers the more realistic route to the clinic. The nNIF-related maturation window likewise remains a developmental hypothesis and requires prospective validation before it can inform the timing of any NET-directed intervention.
